# Genetic flow among olive populations within the Mediterranean basin

**DOI:** 10.7717/peerj.5260

**Published:** 2018-07-11

**Authors:** Valentina di Rienzo, Sara Sion, Francesca Taranto, Nunzio D’Agostino, Cinzia Montemurro, Valentina Fanelli, Wilma Sabetta, Saliha Boucheffa, Abderezak Tamendjari, Antonella Pasqualone, Marion Zammit-Mangion, Monica Marilena Miazzi

**Affiliations:** 1 Department of Soil, Plant and Food Sciences (DISSPA), University of Bari, Bari, Italy; 2 Sinagri s.r.l. Spin-off, Università degli Studi di Bari Aldo Moro, Bari, Italy; 3 Research Centre for Vegetable and Ornamental Crops, CREA, Pontecagnano Faiano (SA), Italy; 4 Laboratoire de Biochimie Appliquée, Faculté des Sciences de la Nature et de la Vie, Université de Bejaia, Bejaia, Algérie; 5 Department of Physiology and Biochemistry, University of Malta, Msida, Malta

**Keywords:** Genetic relationships, Microsatellites, Genetic structure, Wild and cultivated olive trees, Olive, Olive genetic flow, Genetic relationships

## Abstract

**Background:**

The olive tree is a typical crop of the Mediterranean basin where it shows a wide diversity, accounting for more than 2,600 cultivars. The ability to discriminate olive cultivars and determine their genetic variability is pivotal for an optimal exploitation of olive genetic resources.

**Methods:**

We investigated the genetic diversity within 128 olive accessions belonging to four countries in the Mediterranean Basin (Italy, Algeria, Syria, and Malta), with the purpose of better understanding the origin and spread of the olive genotypes across Mediterranean Basin countries. Eleven highly polymorphic simple sequence repeat (SSR) markers were used and proved to be very informative, producing a total of 179 alleles.

**Results:**

Cluster analysis distinguished three main groups according to their geographical origin, with the current sample of Maltese accessions included in the Italian group. Phylogenetic analysis further differentiated Italian and Maltese olive accessions, clarifying the intermediate position of Maltese accessions along the *x*/*y*-axes of principal coordinate analysis (PCoA). Model-based and neighbor clustering, PCoA, and migration analysis suggested the existence of two different gene pools (Algerian and Syrian) and that the genetic exchange occurred between the Syrian, Italian and Maltese populations.

**Discussion:**

The close relationship between Syrian and Italian and Maltese olives was consistent with the historical domestication and migration of olive tree from the North Levant to eastern Mediterranean basin. This study lays the foundations for a better understanding of olive genetic diversity in the Mediterranean basin and represents a step toward an optimal conservation and exploitation of olive genetic resources.

## Introduction

The olive (*Olea europaea* L. subsp. *europaea*, 2*n* = 2*x* = 46) is a primary crop in all the countries of the Mediterranean basin where most of the global production comes from Southern Europe, North Africa, and the Near East (FAOSTAT). The olive germplasm consists of a large number of varieties mainly used for oil or table olive production and each country has a wide panorama of autochthonous cultivated varieties and wild relatives that represents an enormous reservoir of biodiversity and a valuable economic resource (Sardaro et al., 2016). Several studies have been performed to assess genetic diversity as a key action for the valorization of olive genetic resources. Such studies resulted in the description of more than 2,600 cultivars with a wide range of genetic variability in terms of oil content, fruit shape and size, and adaptation to biotic and abiotic stresses (Boucheffa et al., 2017; Khaleghi et al., 2017; Montemurro et al., 2005; Sakar, Hulya & Sezai, 2016). It is well known that olive populations native to the Eastern and Western Mediterranean basin are genetically differentiated most likely because they have adapted to specific environments. The basis of this differentiation is due to gene flow from wild types to cultivated, with the introgression of important alleles from oleaster or from other *O. europaea* subspecies (Besnard et al., 2013; Díez et al., 2015). Information on phylogeny, domestication, and relationships between cultivated and wild forms represents a basic prerequisite for olive breeding (Barazani et al., 2014). Moreover, the recovery of uncommon cultivars is pivotal for preserving the genetic biodiversity from the risk of erosion due to the extensive use of a few elite cultivars (Boucheffa et al., 2017; Rugini et al., 2017). Recently, in different Mediterranean countries, regional projects on biodiversity have led to the establishment of olive germplasm collections for a proper and wide utilization (Díez et al., 2016; Haouane et al., 2011; Muzzalupo, Vendramin & Chiappetta, 2014). This is the first step toward the definition of the role that a certain variety can play in the frame of a sustainable production through its direct use or in breeding programs.

In this research, we focused on accessions deriving from four countries, that is, Italy, Algeria, Syria, and Malta, all characterized by an important olive sector and a rich germplasm heritage adapted to different environmental conditions. In Italy, the Apulia region, which is one of the most important oil producing areas, has wide olive orchards (more than 377,000 ha) spread from the temperate-hot climate coasts to the inner areas at up to 600 m a.s.l., and accounts for more than 50 different varieties, most of which are minor varieties (di Rienzo et al., 2018). Algeria is one of the largest contributors to the Mediterranean oil and table olive production, in particular the region of Kabylie covers 54% of the cultivated area, where the most represented cultivars are Chemlal, Limli, and Azeradj (Boucheffa et al., 2017). Syria is part of the original habitat of *O. europaea*, with 90 cultivars identified so far, although only five varieties, that is, Zaity, Sorani, Doebli, Khoderi, and Kaissy, are extensively cultivated (Al-Ibrahem, Bari & Rashed, 2008; Tubeileh, Abdeen & Al-Ibrahem, 2008). In the Maltese archipelago, three principal cultivars, namely Bidnija, Maltija, and White olive or Bajda, were identified (Mazzitelli et al., 2015) in addition to the rare wild olives characterized by small shrubs, short leaves, and small, bitter-tasting fruits with low oil content. The Maltija variety is highly productive and it is the most common and widespread cultivar in the islands, while Bidnija (from the Bidnija region) is believed to be one of the oldest olive cultivars, indicating that it may date back from the Roman occupation (Buhagiar, 2012). The Bidnija produces oil of excellent quality, is rich in polyphenols and shows high tolerance to environmental stresses such as salinity and drought, and to olive fruit fly (Mazzitelli et al., 2015). “Bajda” produces characteristic white drupes, and it was rediscovered in 2010, as a possible survivor of the famous Maltese “Perlina” or “Pearls of Malta” referenced in Renaissance literature (Verde, 2017). In fact, back in the days of the Crusader Knights of the Order Saint John, known also as the Knights of Malta, who held Malta from 1530 to 1798, the trees carrying these white olives adorned the gardens of the wealthy noblemen (Verde, 2017).

The recently renewed interest, also economical, in the Maltese olive oil sector contrasts with the scarcity of genetic studies carried out on local cultivars (Mazzitelli et al., 2015). Basic questions relating to the migratory movements from which Maltese olive germplasm originate and whether this olive germplasm shows a closed gene pool or an affinity with other Mediterranean countries are still valid (Besnard et al., 2013; Mousavi et al., 2017). In this framework, the purpose of this research was (i) to study the genetic relationships in a collection of 128 wild and cultivated olive accessions from four countries such as Algeria, Syria, Italy, and Malta, including white olives; (ii) to contribute to the enlargement of our knowledge on the genetic differentiation within Mediterranean olive germplasm, and (iii) to help discover the probable origins of Maltese germplasm.

## Materials and Methods

### Samples

A total of 128 olive accessions, both cultivated and wild, were collected from Algeria, Syria, Italy, and Malta (Table 1). The 25 Algerian olive cultivars were sampled from trees in the experimental farm of the Institut Technique de l’Arboriculture Fruitière et de la Vigne (Takarietz, Bejaia, Algeria), located 30 km from Algiers in the Birtouta district. The 16 Algerian accessions, recognized as wild, were selected from different small populations or from isolated trees in different areas in the province of Bejaia (Algeria), where wild and cultivated forms coexist. A total of 33 Syrian accessions were sampled in 2005 from olive trees in the area of Aleppo by the Jussieh Biotechnology Laboratory of the General Commission for Scientific Agricultural Research (Aleppo, Syria). Among the 50 Italian analyzed genotypes, four were collected in private farms from different provinces of the Apulia region (southern Italy) in the frame of a project for the valorization of the Apulian biodiversity (Re.Ger.O.P. project), whereas the remaining were collected in the Pre-Moltiplication field located in Palagiano (Taranto, Italy) in the frame of the OLVIVA project. For the Maltese samples, one was collected from the San-Blas centre, in Zebbug, Malta. The remaining samples were collected from a botanic garden in Attard and from a private garden in Lija, Malta, respectively.

**Table 1:** List of olive accessions collected in different areas of the Mediterranean basin.

Sample no.	*Olea europaea* subsp. *europaea* var.	Accession	Locality	Country	Use
1	*europaea*	Takesrith_BK	Bakaro	Algeria	Oil and table olives
2	*europaea*	Chemlal_SA	SidiAyed	Algeria	Oil
3	*europaea*	Azeradj_SA	SidiAyed	Algeria	Oil and table olives
4	*europaea*	AgrarezIT	Takarietz	Algeria	Oil and table olives
5	*europaea*	Aaleh_IT	Takarietz	Algeria	Oil
6	*europaea*	Akerma_IT	Takarietz	Algeria	Oil and table olives
7	*europaea*	Aberkan_IT	Takarietz	Algeria	Oil and table olives
8	*europaea*	Aghenfas_IT	Takarietz	Algeria	Oil and table olives
9	*europaea*	Abani_IT	Takarietz	Algeria	Oil and table olives
10	*europaea*	Azeradj_IT	Takarietz	Algeria	Oil and table olives
11	*europaea*	Boughenfous_IT	Takarietz	Algeria	Oil
12	*europaea*	Bouichret_IT	Takarietz	Algeria	Oil
13	*europaea*	BouchoukS_IT	Takarietz	Algeria	Oil and table olives
14	*europaea*	BouchoukL_IT	Takarietz	Algeria	Oil and table olives
15	*europaea*	Tabelout_IT	Takarietz	Algeria	Oil
16	*europaea*	Takesrith_IT	Takarietz	Algeria	Oil
17	*europaea*	Tefah_IT	Takarietz	Algeria	Oil and table olives
18	*europaea*	Aberkan_To	Targua Ouzemour	Algeria	Oil and table olives
19	*europaea*	Aharoun_TZ	Tazemalt	Algeria	Oil and table olives
20	*europaea*	Limli_TZ	Tazemalt	Algeria	Oil
21	*europaea*	Chemlal_TZ	Tazemalt	Algeria	Oil
22	*europaea*	Sigoise_TZ	Tazemalt	Algeria	Oil and table olives
23	*europaea*	Azeradj_TZ	Tazemalt	Algeria	Oil and table olives
24	*europaea*	Chemlal_Tdj	Toudja	Algeria	Oil
25	*europaea*	Azeradj_Tdj	Toudja	Algeria	Oil and table olives
26	*sylvestris*	WO_TH	Tala Hamza	Algeria	Wild
27	*sylvestris*	WO1_BK	Bakaro	Algeria	Wild
28	*sylvestris*	WO1_SA	SidiAyed	Algeria	Wild
29	*sylvestris*	WO1_Tdj	Toudja	Algeria	Wild
30	*sylvestris*	WO1_To	TarguaOuzemour	Algeria	Wild
31	*sylvestris*	WO1_TZ	Tazemalt	Algeria	Wild
32	*sylvestris*	WO2_BK	Bakaro	Algeria	Wild
33	*sylvestris*	WO2_SA	SidiAyed	Algeria	Wild
34	*sylvestris*	WO2_Tdj	Toudja	Algeria	Wild
35	*sylvestris*	WO2_To	Targua Ouzemour	Algeria	Wild
36	*sylvestris*	WO2_TZ	Tazemalt	Algeria	Wild
37	*sylvestris*	WO3_SA	SidiAyed	Algeria	Wild
38	*sylvestris*	WO3_Tdj	Toudja	Algeria	Wild
39	*sylvestris*	WO4_SA	Sidi Ayed	Algeria	Wild
40	*sylvestris*	WO5_SA	Sidi Ayed	Algeria	Wild
41	*sylvestris*	WO6_SA	Sidi Ayed	Algeria	Wild
42	*europaea*	Malti Leucocarpa	Zebbug	Malta	Table
43	*europaea*	Tree Malti Lija	Lija	Malta	Oil and table
44	*europaea*	Tree Malti San Anton L	Attard	Malta	Oil
45	*europaea*	Tree Malti San Anton I	Attard	Malta	Ornamental
46	*europaea*	Leucocarpa IT	Puglia	Italy	Oil
47	*europaea*	Oliva bianca IT	Campania	Italy	Oil
48	*europaea*	Leccino	Toscana	Italy	Oil
49	*europaea*	Frantoio	Toscana	Italy	Oil
50	*europaea*	Toscanina	Puglia	Italy	Oil
51	*europaea*	Cima di Melfi	Basilicata	Italy	Oil
52	*europaea*	Nociara	Puglia	Italy	Oil
53	*europaea*	Ascolana tenera	Marche	Italy	Table olives
54	*europaea*	S. Agostino	Puglia	Italy	Table olives
55	*europaea*	Pasola di Andria	Puglia	Italy	Oil
56	*europaea*	Cerasella	Puglia	Italy	Oil and table olives
57	*europaea*	Picholine	Francia	Italy	Oil
58	*europaea*	Cellina di Nardò	Puglia	Italy	Oil
59	*europaea*	Cima di Mola	Puglia	Italy	Oil
60	*europaea*	Coratina	Puglia	Italy	Oil and table olives
61	*europaea*	Carolea	Calabria	Italy	Oil
62	*europaea*	Bella di Cerignola	Puglia	Italy	Oil
63	*europaea*	Cima di Bitonto	Puglia	Italy	Oil
64	*europaea*	Maiatica	Basilicata	Italy	Oil
65	*europaea*	Nocellara del Belice	Sicilia	Italy	Oil
66	*europaea*	Termite di Bitetto	Puglia	Italy	Oil and table olives
67	*europaea*	Donna Giulietta	Puglia	Italy	Oil
68	*europaea*	Ogliarola	Puglia	Italy	Oil
69	*europaea*	Dolce di Cassano	Puglia	Italy	Oil and table olives
70	*europaea*	Cipressino	Puglia	Italy	Oil
71	*europaea*	Ogliarola Garganica	Puglia	Italy	Oil
72	*europaea*	Simona	Puglia	Italy	Oil
73	*europaea*	Pasola	Puglia	Italy	Oil and table olives
74	*europaea*	Nolca	Puglia	Italy	Oil and table olives
75	*europaea*	Olivarossa	Puglia	Italy	Oil
76	*europaea*	Cipressino	Puglia	Italy	Oil
77	*europaea*	Ogliastro	Puglia	Italy	Oil
78	*europaea*	RT1 Moraiolo	Toscana	Italy	Oil
79	*europaea*	RT2 Dritta	Abruzzo	Italy	Oil
80	*europaea*	RT3 Tonda iblea	Sicilia	Italy	Oil
81	*europaea*	RT4 Pendolino	Toscana	Italy	Oil
82	*europaea*	Corniola	Calabria	Italy	Oil
83	*europaea*	Zafarana	Calabria	Italy	Oil
84	*europaea*	Silletta	Puglia	Italy	Oil
85	*europaea*	Grappolo	Puglia	Italy	Oil
86	*europaea*	Frantoiana	Calabria	Italy	Oil and table olives
87	*europaea*	Nostrale	Umbria	Italy	Oil and table olives
88	*europaea*	Coratina_simile	Puglia	Italy	Oil and table olives
89	*europaea*	Ogliarola Salentina	Puglia	Italy	Oil
90	*europaea*	Racioppa	Basilicata	Italy	Oil
91	*europaea*	Rotondella-DPV	Basilicata	Italy	Oil
92	*europaea*	Peranzana-DPV	Puglia	Italy	Oil and table olives
93	*europaea*	Colmona	Puglia	Italy	Oil
94	*europaea*	Pizzutella	Sicilia	Italy	Oil
95	*europaea*	Leucocarpa Pol IT	Puglia	Italy	Oil
96	*europaea*	Mossabi	Southern region	Syria	Oil and table olives
97	*europaea*	Doebli	Coastal region	Syria	Oil and table olives
98	*europaea*	Safrawi	Dar’a	Syria	Oil
99	*europaea*	Sorani	Dar’a	Syria	Oil and table olives
100	*europaea*	Jlot	Dar’a	Syria	Table olives
101	*europaea*	Khodery	Dar’a	Syria	Oil and table olives
102	*europaea*	Mossabi	Aleppo	Syria	Oil and table olives
103	*europaea*	Kaissi yahmoul	Yahmoul	Syria	Table olives
104	*europaea*	Khodery	Idlib	Syria	Oil and table olives
105	*europaea*	Drmalaly	Qmenas	Syria	Oil and table olives
106	*europaea*	Sorani	Yahmoul	Syria	Oil and table olives
107	*europaea*	Mossabi	Qmenas	Syria	Oil and table olives
108	*europaea*	Mossabi	Aleppo	Syria	Oil and table olives
109	*europaea*	Kaissi	Dar’a	Syria	Table olives
110	*europaea*	Zaity	Dar’a	Syria	Oil
111	*europaea*	Safrawi	Dar’a	Syria	Oil
112	*europaea*	Tufahi	Dar’a	Syria	Oil
113	*europaea*	Jlot	Aleppo	Syria	Table olives
114	*europaea*	Mossabi	Yahmoul	Syria	Oil and table olives
115	*europaea*	Sorani	Aleppo	Syria	Oil and table olives
116	*europaea*	Safrawi	Yahmoul	Syria	Oil
117	*europaea*	Doebli	Aleppo	Syria	Oil and table olives
118	*europaea*	Tufahi	Qmenas	Syria	Oil and table olives
119	*europaea*	Zayti	Yahmoul	Syria	Oil
120	*europaea*	Kaissi	Aleppo	Syria	Table olives
121	*europaea*	Kaissi	Dar’a	Syria	Table olives
122	*europaea*	Mousabi	Aleppo	Syria	Wild
123	*europaea*	Jlot shami	Yahmoul	Syria	Wild
124	*europaea*	Jlot shami	Qmenas	Syria	Wild
125	*europaea*	Khoder	Aleppo	Syria	Wild
126	*europaea*	Doebli	Yahmoul	Syria	Wild
127	*europaea*	Khodery	Qmenas	Syria	Wild
128	*europaea*	Dan	Qmenas	Syria	Oil and table olives

Young leaves were collected and immediately frozen. For DNA extraction, 70 mg of lyophilized leaf samples were processed according to Montemurro et al. (2015). DNA quality and concentration were checked using a NanoDrop™ ND2000C (Thermo Fisher Scientific, Waltham, MA, USA); DNA was transferred into a 96-well plate and normalized to a standard concentration of 50 ng/μl by adding HPLC water (Sigma–Aldrich, St. Louis, MO, USA).

### Microsatellite assays

A set of 11 microsatellite markers [simple sequence repeat (SSR)] was selected as the most effective in differentiating the olive accessions (Table S1) (Boucheffa et al., 2017). PCR reactions were performed in a C1000 Touch^TM^ Thermal Cycler (Bio-Rad, Hercules, CA, USA) following the protocol described in Montemurro et al. (2015). In order to verify PCR efficiency, PCR products for each of the 11 SSR markers were randomly checked by electrophoresis on 2.5% SeaKem LE Agarose gel (Lonza, Visp, Switzerland). The amplification products were detected by the automatic sequencer ABI PRISM 3100 Avant Genetic Analyzer (Applied Biosystems, Foster City, CA, USA), and the sample analyses were carried out using the GeneMapper genotyping software v3.7 (Applied Biosystems, Foster City, CA, USA). The internal molecular weight standard was GeneScan^TM^ 500 ROX dye Size Standard (Applied Biosystems, Foster City, CA, USA).

### Statistical analyses for genetic diversity assessment

A total of 11 SSR markers provided clear and unambiguous molecular patterns used to estimate: number of alleles (Na), effective number of alleles (Ne), Shannon’s information index (I), observed (H_o_) and expected (H_e_) heterozygosity, and fixation index (F), using the GENALEX software v.6.5 (http://biology-assets.anu.edu.au/GenAlEx/Welcome.html) (Peakall & Smouse, 2012). The efficiency of each SSR marker to distinguish among the olive accessions was estimated on the basis of allele frequencies by calculating the resolving power (R_p_), which considers the number of polymorphic alleles and the informativeness of a single amplified peak according to Prevost & Wilkinson (1999). Moreover, as additional SSR informativeness, the polymorphic information content (PIC) (Botstein et al., 1980) was calculated by using Cervus v 2.0 (Kalinowski, Taper & Marshall, 2007). The same software was used to estimate the frequency of null alleles.

The analysis of molecular variance (AMOVA) was performed using GenAlex 6.1 in order to estimate the partitioning of the total molecular variance among and within populations. To test the significance of partitioned variance components, *F*-statistic (Wright, 1949) values (Fis, Fit, and Fst) were used with 9,999 permutations for binary data sets (Peakall & Smouse, 2012). GenAlex 6.1 was used also to perform the principal coordinate analysis (PCoA), that gives the inter-individual relationship using Nei’s unbiased genetic distance pairwise population matrix, to determine whether observed patterns in molecular data support the partitioning of the olive samples into specific groupings.

Frequency-based genetic distances were calculated to construct an unweighted neighbor-joining dendrogram for the 128 olive accessions, using DARWIN v 6.0.010 (http://darwin.cirad.fr) (Perrier, Flori & Bonnot, 2003). The resulting tree was bootstrapped with 1,000 replicates (Felsenstein, 1985) and viewed using FigTree 2016-10-04-v1.4.3 available at http://tree.bio.ed.ac.uk/software/figtree/.

Genetic population structure was assessed by using the Bayesian clustering method implemented in the STRUCTURE software version 2.3.4 (https://web.stanford.edu/group/pritchardlab/structure.html) (Pritchard, Stephens & Donnelly, 2000), which assigned accessions in populations (K) based on the Markov Chain Monte Carlo (MCMC) algorithm.

To evaluate the optimal number of populations (K), ten independent runs for each K (from 1 to 10) were performed, using 100,000 MCMC repetitions and 10,000 burn-in periods. Resulting data were analyzed by the Structure Harvester software (Earl & von Holdt, 2012), which is based on ad hoc statistic δ*K* test (Evanno, Regnaut & Goudet, 2005). Accessions were assigned to defined populations if the value of the corresponding membership coefficient (qi) was higher than 0.7, otherwise they were considered to be of admixed ancestry. Based on the groups defined by STRUCTURE analysis, the pairwise *F*_st_ between groups was calculated by using the Genalex software.

In order to infer the phylogenetic relationships and historical admixture events amongst populations, we adopted tree-based approach implemented in TREEMIX (Pickrell & Pritchard, 2012). Firstly, we ran TreeMix on the olive collection, with accessions classified into four populations according to geographical origin. Then, we added ten migration events (*M*) and the *M* value that reached an asymptote and simultaneously provided the smallest residual variance was selected as the most predictive model.

## Results

### Molecular diversity

The genetic variability among 128 Mediterranean olive accessions was analyzed with a set of 11 SSR markers suitable for olive cultivar discrimination (Boucheffa et al., 2017) and the results are showed in Table 2. A total number (Na) of 179 alleles were detected with a mean of 16.27 alleles per locus, ranging from 9 at EMO90 *locus* and 25 at DCA16 *locus*. The number of effective alleles (Ne) *per* SSR ranged from 3.03 (DCA15) to 13.58 (DCA09), with a mean of 7.4. For the same markers, Shannon’s information index (I) ranged from 1.51 (DCA15) to 2.74 (DCA09). The H_o_ ranged between 0.42 for DCA15 and 0.89 for UDO43, whereas the H_e_, which corresponds to heterozygosity at a single *locus* in a theoric panmictic population, ranged between 0.67 (DCA15) and 0.92 (DCA09). In all the accessions under investigation, the mean observed heterozygosity (H_o_ = 0.697) was lower than the mean expected heterozygosity (H_e_ = 0.830), determining a significant positive value for the fixation indices (mean *F* = 0.142) at all *loci* with the exception of UDO43 that showed a negative value (Table 2). The null allele frequencies were lower than 0.20 for the majority of the *loci*, except for DCA15 (0.243). Null allele frequency greater than 0.20 can be considered as a threshold over which a significant underestimation of He can be found (Muzzalupo, Vendramin & Chiappetta, 2014). For this reason, both DCA15 and DCA17 (null allele frequency 0.191) were not considered for downstream analyses.

**Table 2:** Diversity indices of 11 SSR markers detected in a set of 128 olive accessions from Algeria, Italy, Malta, and Syria.

SSR ID	Size range	Na	Ne	I	H_o_	H_e_	F	NAC	R_p_	PIC	F *nulli* (%)
DCA03	232–257	12	8.24	2.24	0.87	0.87	0.009	30	2.33	0.87	0.37
DCA05	194–220	11	6.25	2.00	0.72	0.84	0.138	28	2.02	0.82	7.17
DCA09	162–210	20	13.58	2.74	0.81	0.92	0.116	47	2.18	0.92	6.07
DCA13	110–156	13	3.67	1.74	0.58	0.72	0.194	22	2.00	0.70	12.70
DCA15	246–275	10	3.03	1.51	0.42	0.67	0.371	16	3.06	0.68	24.25
DCA16	120–191	25	11.21	2.71	0.87	0.91	0.038	45	2.00	0.91	1.88
DCA17	107–189	23	5.36	2.16	0.55	0.81	0.321	34	2.01	0.80	19.09
DCA18	116–207	19	7.72	2.29	0.80	0.87	0.081	39	2.10	0.86	4.30
UDO43	170–222	22	9.27	2.58	0.89	0.89	−0.007	39	2.02	0.89	0.95
GAPU101	180–219	15	7.18	2.18	0.75	0.86	0.121	32	2.01	0.84	6.58
EMO90	182–202	9	6.09	1.91	0.68	0.83	0.185	23	2.35	0.82	10.70
Total		179									
Mean		16.27	7.4		0.72	0.830	0.142			0.82	

**Note:**

Na, number of observed alleles; Ne, effective alleles; I, Shannon’s information index; H_o_, observed heterozygosity; H_e_, expected heterozygosity; F, fixation index; NAC, number of allele combinations; R_p_, resolving power; PIC, polymorphic information content; F *nulli*, frequency of *nulli* alleles.

The number of allele combinations ranged from 16 at the *locus* DCA15 to 47 at *locus* DCA09 (Table 2). The efficiency of the SSR markers in distinguishing the accessions was estimated calculating the *R_p_* and the PIC indices. Both indices indicated a powerful discrimination ability of markers. In fact, *R_p_* ranged from 2.00 (DCA13 and DCA16) to 3.06 (DCA15) (Table 2). PIC values were between 0.68 and 0.92 for DCA15 and DCA09, respectively, with an average of 0.82, indicating that all *loci* were highly informative (PIC > 0.50).

### Genetic diversity analysis

**Figure 1:**
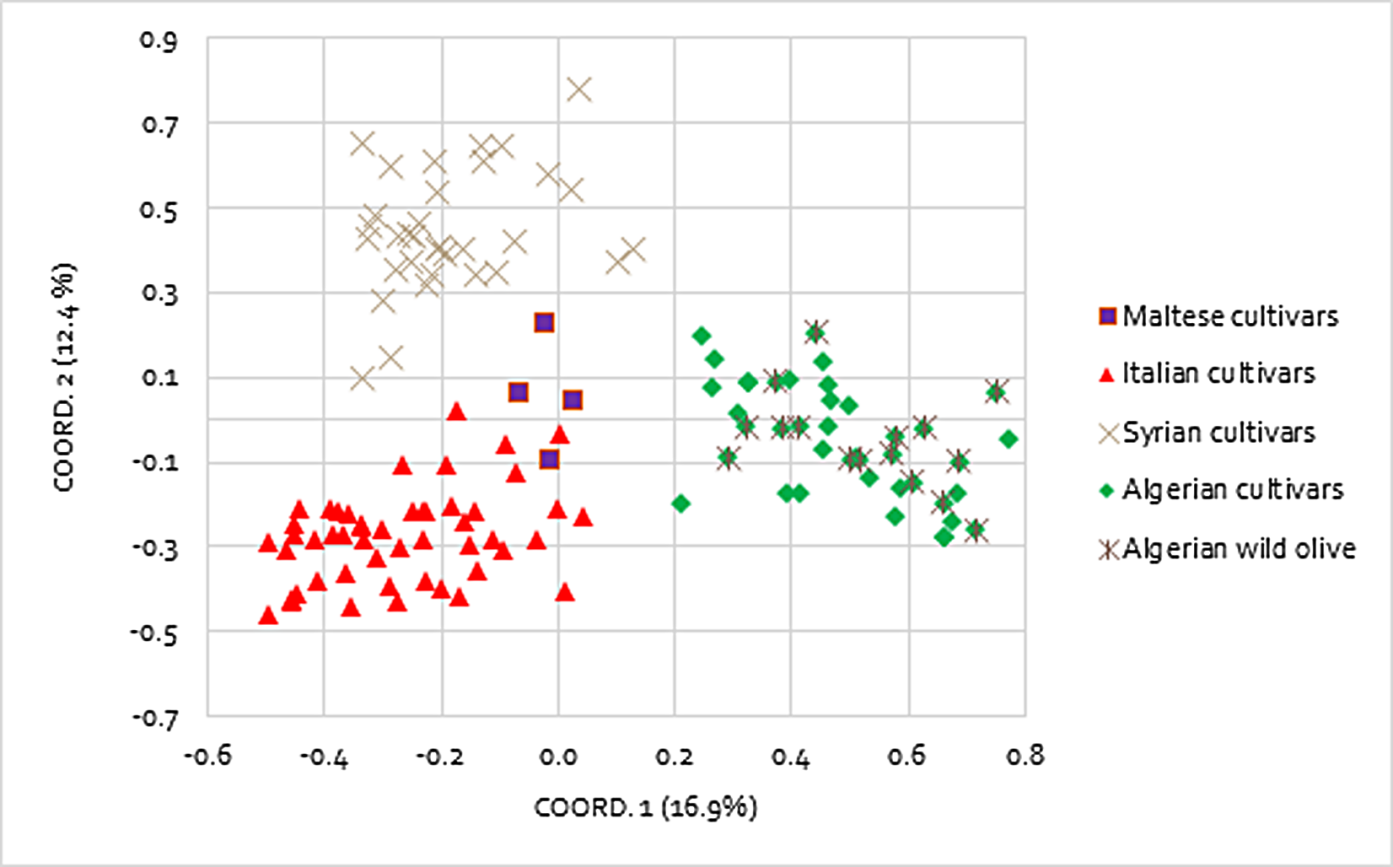
Principal coordinates analysis (PCoA). Differentiation among 128 Mediterranean olive accessions based on nine polymorphic SSR markers.

The genetic relationships between the Mediterranean olive accessions were investigated by using PCoA performed on Nei’s unbiased genetic distance matrix (Fig. 1). A total of four different groups were obtained corresponding to the geographical area of origin: Italy, Malta, Algeria, and Syria. The first (PCo1) and the second principal coordinates (PCo2) explained 16.99% and the 12.44% of the variance in the molecular data, respectively. In particular, the PCo2 clearly discriminated the Syrian genotypes from the Italian ones, whereas PCo1 separated the Algerian accessions from the remaining ones. The Maltese samples remained in the middle between the Italian and Syrian genotypes and all of them were very distant from the Algerian. The AMOVA analysis assigned most of the molecular variance to individuals (73%) and only 12% and 15% among individuals and among the four groups, respectively (Table 3). The *F*-statistic test, that relates the diversity within-population to the total genetic diversity, confirmed the significance of the partitioned variance components, with values of *F*_st_ = 0.152, *F*_it_ = 0.268, and *F*_is_ = 0.137.

**Table 3:** Analysis of molecular variance (AMOVA)*.

Source of variance	Df	Sum Sq	Mean Sq	Variance components	%	*P*
Among groups	3	157.540	52.513	0.819	15.0	<0.001
Among individuals	124	642.343	5.180	0.623	12.0	<0.001
Within individuals	128	503.500	3.934	3.934	73.0	<0.001
*F*-statistics	Value					
*F*_st_ (among populations)	0.152					
*F*_is_ (within populations)	0.137					
*F*_it_ (total)	0.268					
*F*_st_ max	0.241					
*F*’_st_	0.632					

**Notes:**

*P*-value is based on 1,000 permutations.

Df, degree of freedom; SS, sum of squares; MN, mean squares; %, percentage of total variation.

* The partitioning of genetic variation within and between groups obtained with PCoA, and *F*-statistic values for the 128 olive accessions collected from Algeria, Italy, Malta, and Syria.

### Genetic structure of Mediterranean olive genotypes

**Figure 2:**
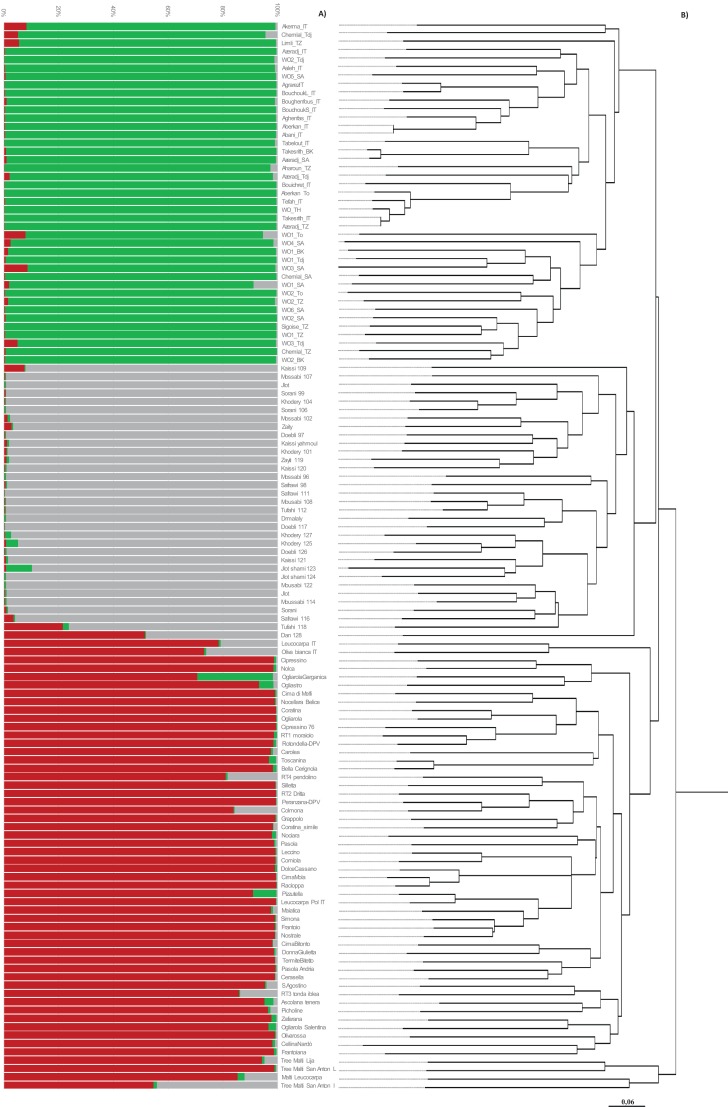
Genetic structure and phylogenetic tree of Mediterranean olive genotypes. (A) Bar plot showing clusters inferred by STRUCTURE. Each vertical line stands for a single accession and it is divided into K colored segments that represent the estimated membership coefficient (q). Maltese accessions are grouped with Italian genotypes. (B) Neighbor-Joining dendrogram obtained with DARWIN v 6.0.010.

Population structure was investigated by the Bayesian-based STRUCTURE analysis. The analysis showed a clear maximum for Δ*K* at *K* = 3 and as result all accessions were grouped into three different populations, with four accessions assigned to the admixed group (Fig. 2A). Populations could be discriminated to a great extent on the basis of the geographical origin. In more detail, population 1 is comprised of 41 accessions from Algerian cultivars and wild oleasters; population 2 includes all the 32 Syrian accessions, unless sample Dan 128 that falls in the admixed group. Population 3 groups 50 Italian accessions along with the Maltese cultivars (Maltija San Anton Inner, Malti Leucocarpa, and Tree Malti Lija), with the exception of Maltija San Anton, which shares admixed allele frequencies. A good differentiation among groups was also indicated by pairwise *F*_st_ estimates among the three groups, thus confirming a territorial distinctiveness of the gene pools. Indeed, *F*_st_ value was 0.088 between population 1 (Algeria) and population 3 (Italy), *F*_st_ was 0.122 between population 1 (Algeria) and population 2 (Syria), and *F*_st_ was 0.079 between population 2 (Syria) and population 3 (Italy). H_o_, H_e_, and the fixation index (F) were also calculated within each group (Table S2).

For population 1, the mean values were 0.805 (H_o_), 0.779 (H_e_), and −0.037 (*F*); for population 2: 0.772 (H_o_), 0.801 (H_e_), 0.038 (*F*); for population 3: 0.771 (H_o_), 0.713 (H_e_), and −0.075 (*F*).

The neighbor-joining dendrogram partially supports the results from population structure analysis, showing high to moderate differentiation within the olive collection with a total of four groups, attributable to common geographical origin (Fig. 2B). A first node separates Maltese accessions from the remaining ones, which in turn are divided by a second node: Algerian and Syrian olives from Italian germplasm. Cluster 1 consists of 41 Algerian accessions that are divided into two distinct branches depending on whether they are cultivated varieties or wild oleasters. The wild accessions WO2_Tdj, WO5_SA, and WO_TH cluster along with the cultivated accessions, while the cultivated varieties Chemlal_Sa, Chemlal_Tz, and Sigoise_TZ group with the wild accessions (Fig. 2B). This suggests that wild olive genotypes are strictly genetically related to cultivated germplasm and may represent feral forms resulting from gene flow between local cultivars and oleaster genotypes, as expected in areas where the two botanical varieties share a common environment with the oleaster trees located in close proximity to the cultivated fields (Boucheffa et al., 2017). Cluster 2 is composed by 31 Syrians accessions. Kaissi 109 (Southern Syria) and Dan 128 (Northern Syria) clearly split out of the group. A total of five out of six wild accessions, named Jlot shami (123 and 124), Khoder_125, Doebli_126, and Khodery_127, all collected from the Northern Syrian areas (Aleppo, Dar’a, Yahmoul, and Qmenas), were clustered together in the same subgroup along with the table olive variety Kaissi, thus suggesting a common genetic background. One exception was represented by the wild accession Mousabi_122, showing relatedness with other Syrian cultivars.

Cluster 3 included the Italian varieties originated from Abruzzo, Apulia, Basilicata, Calabria, Campania, Marche, Sicily, and Tuscany. Different cases of homonymy have been identified. Olive trees under the “Cipressino_70” and “Cipressino_76” denomination (Apulia region) were classified into two molecular profiles and were different at eight SSR alleles; “Ogliarola_68” and “Ogliarola Garganica_71” (Apulia region) were differentiated by 10 SSR alleles; “Ogliarola_68” and “Ogliarola Salentina_89” were differentiated by three SSR alleles, whereas “Ogliarola Garganica_71” and “Ogliarola Salentina_89” were differentiated by nine SSR alleles. “Coratina_60” and “Coratina_simile_88” (Apulia region) were differentiated by 11 SSR alleles.

The rare white cv. Oliva Bianca IT and Leucocarpa IT clustered together and were clearly separate from the other Italian accessions such as LeucoarpaPal, indicating that the probability of the same mutation affecting anthocyanin synthesis, responsible for the white color of ripened olives, occurred in different accessions (Pasqualone et al., 2012). Moreover, these white cultivars were genetically distinct from Leucocarpa Malti (Bajda). A strong relationship was found between cultivar Toscanina and Bella di Cerignola (both from Apulian region), and between Cima di Mola (Apulia region) and Racioppa (Basilicata region). In the last case, the two adjacent regions might provide evidence for the movement and exchange of germplasm. A group of varieties characterized by both high fruit weight and table use of the drupes clustered close in two subgroups originating from cluster 3: Termite di Bitetto, Pasola di Andria, and Cerasella on one side, and Sant’Agostino, Tonda Iblea, Ascolana Tenera, and Picholine on the other. Cluster 4 includes four Maltese accessions, consisting of cultivated (Leucocarpa) and (Malti-Lija) and wild oleaster (Malti San Anton L and Malti San Anton I).

**Figure 3:**
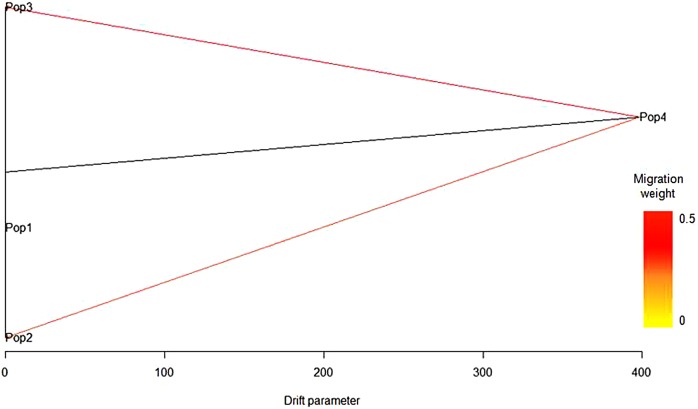
Predictive model based on log-likelihood and residual variance values obtained with TreeMix. The first migration event was predicted by TreeMix software from Syrian population (Pop4) toward the Italian population (Pop3) and a second migration edge was directed from the Syrian population (Pop4) to the Maltese population (Pop2). No migration events occurred between the Algerian population (Pop1) and the others.

Based on log-likelihood and residual variance values, the most predictive model suggested the presence of two migration events (Fig. 3). The significant migration edge with the highest weight (0.47) was directed from the root of Syrian population (Pop4) toward the Italian population (Pop3). A second migration edge (0.36) was directed from Syrian population (Pop4) to the root of the Maltese population (Pop2). No migration events occurred between the Algerian population and the others.

## Discussion

Mediterranean landscape and culture has in the olive tree its distinctive element, and olive oil production is among the sectors of high economic significance in the area. Indeed, olive tree cultivation and marketing of olive oil and table olives are major sources of employment and income in the Mediterranean Basin. For these reasons, many projects addressing the characterization, conservation and utilization of olive genetic resources have been recently funded by local administrations in several Mediterranean countries (Sardaro et al., 2016). Genetic diversity represents a heritage of high scientific value and the availability of autochthonous germplasm can help to improve the long-term productivity potential of olive orchards and enhance the competitiveness of the sector in a globalized market. Together the four Mediterranean countries considered in this research, that is, Italy, Malta, Algeria, Syria, own valuable reservoirs of olive germplasm that are largely unexploited in terms of morphological, phenological, bio-agronomical, and productive traits (Tubeileh, Abdeen & Al-Ibrahem, 2008; Linos et al., 2014). Successful breeding programs for yield and quality require deep knowledge on the genetic diversity of the available germplasm that provide also insights into the ability of the species to cope with environmental changes. A detailed and unequivocal characterization of the germplasm cannot be achieved through only morphological descriptions, whereas molecular markers, such as microsatellites, still allow a more precise determination of cultivars (Díez et al., 2011; Erre et al., 2010; Fendri et al., 2014). Indeed, SSRs have being extensively used in genetic studies, marker-assisted selection, cultivar identification, and varietal traceability of olive oil and table olives (Pasqualone et al., 2016) due to their versatility in providing a quick assay and for their high informativeness related to high repeatability, codominant nature, specificity, and multiple alleles (Cheng et al., 2009; Sakar, Hulya & Sezai, 2016).

**Figure 4:**
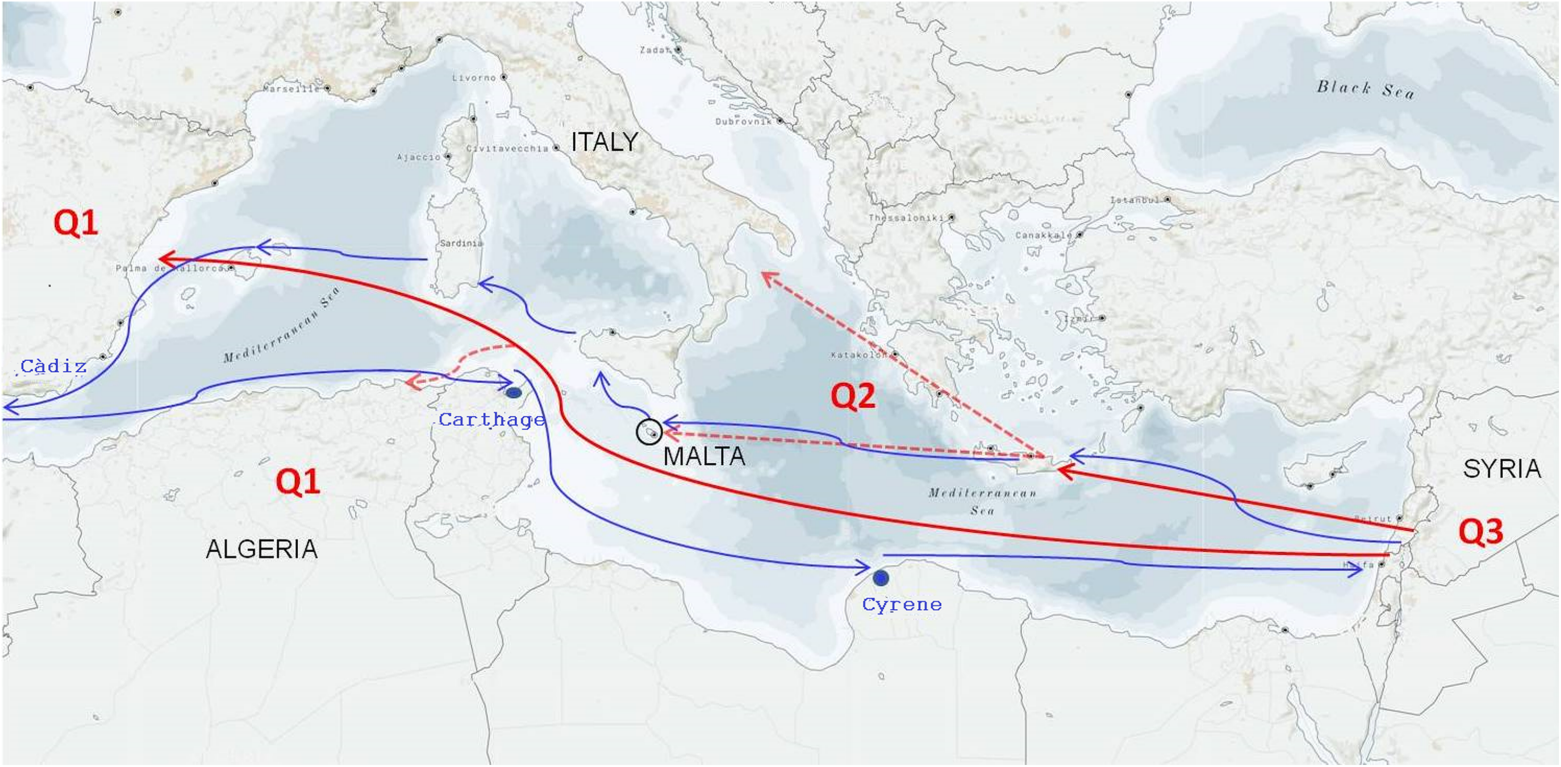
Hypothesis of the primary domestication and secondary diversification of the olive in the Mediterranean Basin. Q1, Q2, and Q3 represent the three main olive gene pools matching the Western, Central, and Eastern geographical areas, respectively (Besnard et al., 2013; Díez et al., 2015). The red continuous arrows describe the migration of olives from Syria to the Greek area and the secondary independent event of domestication from Syria to Spain. The red dotted arrows indicate that a second migration event occurred in Italy and Malte from the Greek area and in Algeria from Spain. The blue arrows retrace the ancient Phoenician navigation routes.

The goal of our research was to shed light on the genetic relationships of 128 varieties, including wild accessions, from four countries of the Mediterranean Basin. This was achieved using the best possible set of SSR markers retrieved from recent literature on the topic (Boucheffa et al., 2017; Fernández i Martí et al., 2015). Besides, the majority of the microsatellites used were proposed by a collaborative study between four independent laboratories for high power of discrimination and reproducibility due to low peak stuttering, strong peak signal, and absence of null alleles (Baldoni et al., 2009). The markers were confirmed to be very effective, showing high Rp and PIC. The values of Rp were in the range observed in a previous work (Pasqualone et al., 2013). In particular, DCA09 and DCA16 produced the highest number of allelic combinations and number of distinguished accessions. Across the 128 analyzed olive accessions, a certain genetic diversity was detected, but it was lower than that indicated in olives grown in the Mediterranean area in similar works (Abdessemed, Muzzalupo & Benbouza, 2015; Fernández i Martí et al., 2015; Muzzalupo, Vendramin & Chiappetta, 2014). High values of heterozygosity are expected in olive, a species that is mainly propagated via vegetative growth, but that is subjected to natural crossing (olive tree is allogamous), somatic mutation events that contribute to expand its genetic variability (di Rienzo et al., 2018; Martins-Lopes et al., 2009). In our collection, we obtained positive fixation indices at all SSR markers (except at *locus* UDO43), indicating a defect of heterozygosity in the collection. Díez et al. (2016) described higher values of H_o_ over H_e_ and other authors have reported a defect of heterozygosity in olive, ascribing it to differences in plant samples and in the set of genetic markers (Lumaret et al., 2004; Rugini et al., 2017), resulting in numerous null alleles (Erre et al., 2010), exactly like in this study. In fact, even removing the *loci* with *nulli* allele frequencies >20, the heterozygosity remained at a low level. Moreover, we found an excess of heterozygosity in the Algerian (cluster I) and Italian (cluster III) accessions, but not in the Syrian (cluster II) accessions. This result could be due to the limited area of origin of the Syrian accessions, and to the selection operated on some alleles (Lumaret & Ouazzani, 2001). Regarding the clustering, the Bayesian analysis grouped accessions into three main gene pools, clearly corresponding to their geographical origin Algeria, Italy and Malta, and Syria. By contrast, in the dendrogram, the split of Maltese accessions at 0.15 of similarity index from the rest of the accessions under investigation is evident, thus supporting the hypothesis of a local differentiation, as already reported by Mazzitelli et al. (2015) and as occurred in Cyprus island (Anestiadou et al., 2017), even though the number of genotypes is small and the Maltese germplasm will require more investigation. We detected two migration events, which are consistent with gene flow that occurred between Syrian, Italian, and Maltese populations and allow to speculate about olive differentiation. The most well-substantiated hypothesis on the origin and spread of cultivated olive trees across the Mediterranean basin is based on the existence of three main genetic pools that match the geographical areas of West (namely Q1), Centre (Q2), and East (Q3) of the Mediterranean basin (Díez et al., 2015, 2016), where olive cultivation developed around 5,000 years ago (Breton et al., 2009; Belaj et al., 2012; Besnard et al., 2013; Chalak et al., 2014). We suggested a probable scenario about the origin and spread of olive germplasm under study. Considering that Italian and Maltese accessions shared the same allelic frequencies and the Maltese accessions are genetically distant from the others in the dendrogram, two main gene pools might be present in our collection. The first gene pool includes only the Algerian accessions, whereas the second gene pool comprises Syrian, Italian and Maltese accessions. It is interesting to observe that both Italian and Maltese population seem to derive from the Syrian population, probably before the Roman colonization and dating back to the navigation routes made by the Phoenicians (Fig. 4). In fact, historically the Phoenicians came from the Lebanese seacoast, at the edge with the modern Syria, which is considered the place where the first domestication of olive tree occurred (Fig. 4). Therefore, the same allelic frequencies between Italian and Maltese accessions can arise from the common Syrian ancestor. Overall, our results showed that each country is characterized by a particular gene pool and this is in agreement with many studies on the genetic diversity of cultivated olive, which indicate how critical the geographical origin is in determining the grouping of accessions on a genetic basis (Besnard et al., 2013; Biton et al., 2015; Marra et al., 2013; Yoruk & Tuskin, 2014; Taranto et al., 2018; D’Agostino et al., 2017).

## Conclusion

The use of SSRs has proved useful for the detection of genetic differences and relationships among the Mediterranean olive cultivars, confirming that each country has a germplasm that needs to be preserved and valued. Our research, studying the genetic relationships in a collection of 128 wild and cultivated olive accessions from Algeria, Syria, Italy, and Malta, contributes to the enlargement of our knowledge on the genetic differentiation within Mediterranean olive germplasm.

## Supplemental Information

10.7717/peerj.5260/supp-1 Supplemental Information 1 Table S1. List of the 11 microsatellite markers (SSR) tested on olive accessions. For each SSR, the identification code (SSR ID), bibliographic reference, repeat motif, primer sequence and annealing temperature (Ta) is reported.

10.7717/peerj.5260/supp-2 Supplemental Information 2 Table S2. Genetic diversity parameters at SSR loci estimated in the three groups identified following population structure analysis. For each cluster, the observed heterozygosity (Ho), the expected heterozygosity (He), and the fixation index (F) are reported. Cluster I (Algerian accessions), Cluster II (Syrian accessions), Cluster III (Italian accessions).

10.7717/peerj.5260/supp-3 Supplemental Information 3 Raw data and statistics.

## Additional Information and Declarations

### Data Availability

The following information was supplied regarding data availability: The raw data are provided in a Supplemental File.
