# Supplementary material for: Genetic flow among olive populations within the Mediterranean basin
Source: PeerJ. 2018 Jul 11;6:e5260. doi: 10.7717/peerj.5260 (PMC6045921; doi:10.7717/peerj.5260)
Supplement: Supplemental Information 1 — For each SSR, the identification code (SSR ID), bibliographic reference, repeat motif, primer sequence and annealing temperature (Ta) is reported. [file peerj-06-5260-s001.docx]

**Supplementary Table (S1). List of the 11 microsatellite markers (SSR) tested on olive accessions.** For each SSR, the identification code (SSR ID), bibliographic reference, repeat motif, primer sequence and annealing temperature (Ta) is reported.

| SSR ID | Bibliographic reference | Repeat  motif | Primer Sequence (5'-3') | Ta |
| --- | --- | --- | --- | --- |
| DCA03 | Sefc *et al*. (2000) | (GA)_19_ | cccaagcggaggtgtatattgttac | 50°C |
|  |  |  | tgcttttgtgtttgagatgttg |  |
| DCA05 | Sefc *et al*. (2000) | (GA)_15_ | aacaaatcccatacgaactgcc | 50°C |
|  |  |  | cgtgttgctgtgaagaaaatcg |  |
| DCA09 | Sefc et al. (2000) | (GA)_23_ | aatcaaagtcttccttctcatttcg | 55°C |
|  |  |  | gatccttccaaaagtataacctctc |  |
| DCA13 | Sefc et al. (2000) | (CA)_15_ | gatcagattaatgaagatttggg | 55°C |
|  |  |  | aactgaacctgtgtatcttgcatcc |  |
| DCA15 | Sefc et al. (2000) | (CA)_3_G(AC)_14_ | gatcttgtctgtatatccacac | 50°C |
|  |  |  | tataccttttccatcttgacgc |  |
| DCA16 | Sefc et al. (2000) | (GT)_13_(GA)_29_ | ttaggtgggattctgtagatggttg | 50°C |
|  |  |  | ttttaggtgagttcatagaattagc |  |
| DCA17 | Sefc et al. (2000) | (GT)_9_(AT)_7_AGATA(GA)_38_ | gatcaaattctaccaaaaatata | 50°C |
|  |  |  | taatttttggcacgtagtattgg |  |
| DCA18 | Sefc et al. (2000) | (CA)_4_CT(CA)_3_(GA)_19_ | aagaaagaaaaaggcagaattaagc | 50°C |
|  |  |  | gttttcgtctctctacataagtgac |  |
| UDO43 | Cipriani et al. (2002) | (GT)_12_ | tcggctttacaacccatttc | 57°C |
|  |  |  | tgccaattatggggctaact |  |
| GAPU101 | Cipriani et al. (2002) | (GA)_8_(G)_3_(AG)_3_ | catgaaaggagggggacata | 57-60°C |
|  |  |  | ggcacttgttgtgcagattg |  |
| EMO90 | De la Rosa et al. (2002) | (CA)_10_ | catccggatttcttgctttt | 50°C |
|  |  |  | agcgaatgtagctttgcatgt |  |
