# Supplementary material for: Genetic flow among olive populations within the Mediterranean basin
Source: PeerJ. 2018 Jul 11;6:e5260. doi: 10.7717/peerj.5260 (PMC6045921; doi:10.7717/peerj.5260)
Supplement: Supplemental Information 2 — For each cluster, the observed heterozygosity (Ho), the expected heterozygosity (He), and the fixation index (F) are reported. Cluster I (Algerian accessions), Cluster II (Syrian accessions), Cluster III (Italian accessions). [file peerj-06-5260-s002.docx]

**Supplementary Table (S2)**

**Genetic diversity parameters at SSR loci estimated in the three groups identified following population structure analysis**. For each cluster, the observed heterozygosity (Ho), the expected heterozygosity (He), and the fixation index (F) are reported. Cluster I (Algerian accessions), Cluster II (Syrian accessions), Cluster III (Italian accessions).

| Cluster | SSR ID | Ho | He | F |
| --- | --- | --- | --- | --- |
| Cluster I | DCA03 | 0.878 | 0.831 | -0.057 |
|  | DCA05 | 0.732 | 0.720 | -0.017 |
|  | DCA09 | 0.868 | 0.863 | -0.006 |
|  | DCA13 | 0.846 | 0.815 | -0.038 |
|  | DCA16 | 0.951 | 0.869 | -0.095 |
|  | DCA18 | 0.850 | 0.799 | -0.064 |
|  | UDO43 | 0.878 | 0.872 | -0.007 |
|  | GAPU101 | 0.683 | 0.740 | 0.077 |
|  | EMO90 | 0.561 | 0.499 | -0.123 |
|  | **Mean** | **0.805** | **0.779** | **-0.037** |
| Cluster II |  |  |  |  |
|  | DCA03 | 0.833 | 0.789 | -0.056 |
|  | DCA05 | 0.741 | 0.796 | 0.069 |
|  | DCA09 | 0.698 | 0.885 | 0.211 |
|  | DCA13 | 0.547 | 0.650 | 0.158 |
|  | DCA16 | 0.889 | 0.843 | -0.055 |
|  | DCA18 | 0.796 | 0.846 | 0.058 |
|  | UDO43 | 0.902 | 0.823 | -0.096 |
|  | GAPU101 | 0.833 | 0.858 | 0.028 |
|  | EMO90 | 0.704 | 0.721 | 0.024 |
|  | **Mean** | **0.772** | **0.801** | **0.038** |
| Cluster III |  |  |  |  |
|  | DCA03 | 0.931 | 0.719 | -0.294 |
|  | DCA05 | 0.697 | 0.544 | -0.282 |
|  | DCA09 | 1.000 | 0.857 | -0.167 |
|  | DCA13 | 0.310 | 0.524 | 0.407 |
|  | DCA16 | 0.720 | 0.677 | -0.064 |
|  | DCA18 | 0.750 | 0.825 | 0.091 |
|  | UDO43 | 0.962 | 0.734 | -0.310 |
|  | GAPU101 | 0.714 | 0.778 | 0.082 |
|  | EMO90 | 0.857 | 0.756 | -0.133 |
|  | **Mean** | **0.771** | **0.713** | **-0.075** |
